# The Enhancement of Biomass Accumulation, Caffeoylquinic Acid Derivative Production, and Antioxidant Activity of *Rhaponticum carthamoides* Transformed Roots Cultured in a Nutrient Sprinkle Bioreactor

**DOI:** 10.3390/ijms26041422

**Published:** 2025-02-08

**Authors:** Ewa Skała, Monika A. Olszewska, Agnieszka Kicel

**Affiliations:** 1 Department of Biology and Pharmaceutical Botany, Medical University of Lodz, Muszynskiego 1, 90-151 Lodz, Poland; 2 Department of Pharmacognosy, Medical University of Lodz, Muszynskiego 1, 90-151 Lodz, Poland; monika.olszewska@umed.lodz.pl (M.A.O.); agnieszka.kicel@umed.lodz.pl (A.K.)

**Keywords:** nutrient sprinkle bioreactor, PlantForm, Rita, temporary immersion system

## Abstract

*Rhaponticum carthamoides* (Willd.) Iljin. is an endemic plant species found in Siberia, Mongolia, and Kazakhstan. Its roots and rhizomes are used to treat physical fatigue and weakness following illness. The present study examines the scaling up of caffeoylquinic acid (CQA) derivative and flavonoid production in *R. carthamoides* transformed roots. The transformed roots were grown in shaken Erlenmeyer flasks of varying volumes (0.5–2 L), a temporary immersion system (TIS) (Rita^®^ and PlantForm bioreactors), and a nutrient sprinkle bioreactor (NSB) in Woody Plant medium for 35 days. The highest dry biomass production was achieved in the 0.5 L and 1 L flasks and in the NSB bioreactor, yielding 22.2 to 20.4 g/L—approximately 14 to 23 times the weight of the inoculum. The accumulation of individual specialized metabolites varied depending on the culture system used. The peak amount of CQAs (544.5 mg/L), in terms of the increase in dry weight and metabolite levels, was obtained in the NSB bioreactor. The primary CQAs were chlorogenic acid (5-CQA) and a tri-CQA 1. The highest concentration of 5-CQA (7.38 mg/g DW) was found in the roots cultivated in the NSB bioreactor. In contrast, the tri-CQA 1 dominated in the roots from 2 L shaken Erlenmeyer flasks (8.44 mg/g DW). Our findings demonstrate that transformed roots growing in an NSB bioreactor are an effective system for increasing CQA production, potentially serving as an alternative source. This biotechnological approach could help reduce the overexploitation of field-grown *R. carthamoides*, a currently threatened species.

## 1. Introduction

Plants have been used for thousands of years for functional or medicinal purposes. Their biological activity is attributed to the various specialized metabolites they produce, which enable protection against environmental stress, pathogens, and herbivores, attract pollinators, and enhance their survival through allelopathy [1,2,3,4]. Plants are rich sources of various active substances, including polyphenols, terpenoids, alkaloids, and polyacetylenes; many have considerable biological value due to their antioxidant, antimicrobial, and anti-inflammatory properties, as well as their anticancer, adaptogenic, or anabolic potential. Incorporating specialized plant metabolites into the daily diet plays an important role in preventing numerous lifestyle-related diseases, including diabetes, cancer, and cardiovascular diseases. As a result, there is a significant demand for plant metabolites in the pharmaceutical, cosmetic, and food industries, where they are used as natural preservatives, stabilizers, dyes, and flavourings.

There is also great interest in identifying new sources of active plant metabolites for creating functional plant-based diets and producing valuable biopharmaceuticals, dietary supplements, herbal medicines, and natural cosmetics. However, excessive harvesting of specialized metabolites from plants in their natural habitats may lead to extinction. Therefore, new methods for obtaining specialized metabolites are under investigation. One particularly promising area is in vitro plant culture, such as transformed roots obtained through genetic transformation using *Rhizobium rhizogenes* soil bacteria. The transformed roots exhibit rapid growth and biomass increases in a short period. Additionally, they are autotrophic to growth regulators and often accumulate higher levels of valuable specialized metabolites than soil-grown plant roots [5,6]. Such production may be further enhanced by various strategies, such as the selection of clones, culture conditions (e.g., medium type, carbon source, light condition), elicitor treatment, precursor feeding, or metabolic engineering of the regulatory genes in metabolic pathways [7,8].

*Rhaponticum carthamoides* (Willd.) Iljin. (Maral root) is an endemic plant species naturally distributed in Siberia, Kazakhstan, Mongolia, and China [9,10,11]. Maral root has been used in traditional medicine to alleviate physical fatigue and weakness following illness [9]. The roots and rhizomes of *R. carthamoides* are included in official pharmacopoeias of some Eastern European countries [10]. Our previous studies have shown that *R. carthamoides* transformed roots are a rich source of bioactive caffeoylquinic acid derivatives (CQAs) [12,13], known to display a wide range of pharmacological activities [14]. By selecting root clones and optimizing growth medium, light conditions, and sucrose concentration, it was possible to obtain greater production of CQAs in *R. carthamoides* transformed roots compared to the soil-grown plant roots [12,13]. In vitro studies revealed that the aqueous methanol extract from *R. carthamoides* transformed roots exhibited cytotoxic and genotoxic effects and induced apoptosis in various cancer cell lines [15,16,17].

The current study aims to scale up production of CQAs by culturing *R. carthamoides* transformed roots in Woody Plant medium (WPM) in Erlenmeyer flasks of various volumes (0.5–2 L) and in two types of bioreactors: a temporary immersion system (TIS) (Rita^®^ and PlantForm) and a nutrient sprinkle bioreactor (NSB) with a 5 L capacity for 35 days. Furthermore, the antioxidant potential of *R. carthamoides* transformed roots was evaluated in vitro using a non-cellular model of various reactive oxygen species (ROS) associated with systemic oxidative stress in vivo.

## 2. Results and Discussion

*R. carthamoides* is an endemic plant species threatened with extinction due to the overexploitation of its roots and rhizomes in their natural habitat [18]. However, its raw materials may also be obtained by in vitro plant culture, which can be scaled up to facilitate the large-scale biosynthesis of specialized metabolites in bioreactors [7]. Therefore, the aim of the present study was to evaluate the potential of *R. carthamoides* transformed roots for large-scale cultivation.

To this end, the study compares the effects of cultivation in Erlenmeyer flasks of various volumes (500 mL, 1 L, and 2 L) using a rotary shaker and two types of bioreactors: (a) a temporary immersion system (PlantForm and Rita^®^) and (b) a nutrient sprinkle bioreactor. In all cases, optimized culture conditions were provided, consisting of WPM medium supplemented with 3% sucrose in the light [12,13]. To achieve high root biomass growth and efficient production of desired specialized metabolites in the bioreactors, it is crucial to identify several key factors, such as selecting an appropriate bioreactor with the right substrate supply system and ensuring adequate culture aeration.

### 2.1. Growth Characteristics of R. carthamoides Transformed Roots

The transformed root cultures are mainly grown on a small laboratory scale in flasks on a shaker. Such flask culture can restrict nutrient and oxygen access, leading to uneven growth and tissue necrosis [19]. The root cultures can be performed in bioreactors to reduce cultivation costs and increase the efficiency of root biomass and specialized metabolite production.

**Figure 1:**
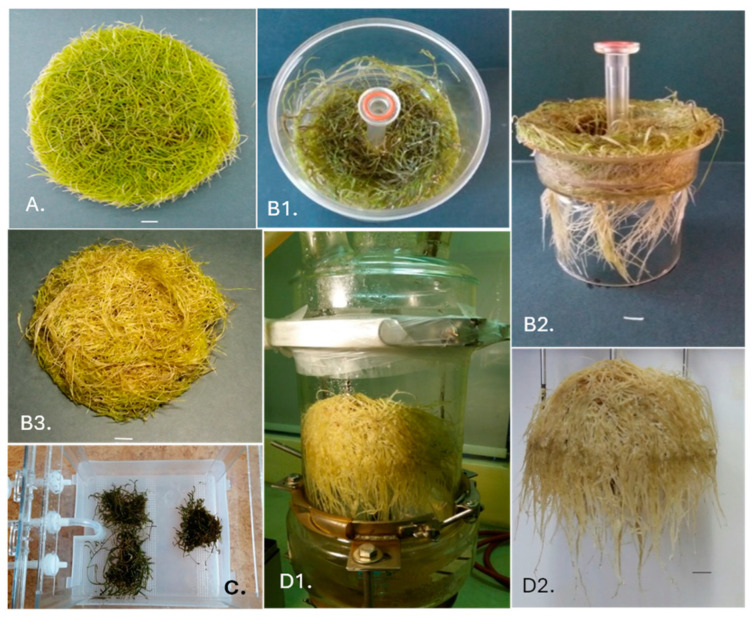
*R. carthamoides* transformed roots after 35 days of cultivation in liquid WPM medium in a 1 L flask on the rotary shaker (**A**), temporary immersion bioreactor Rita^®^ (**B1**–**B3**) and PlantForm* (**C**), and nutrient sprinkle bioreactor (NSB) (**D1**,**D2**). * The cultures were grown for 21 days. Scale bar = 1 cm.

The present study is the first to compare *R. carthamoides* transformed root growth in shake flasks and bioreactors. The obtained roots were green, with the colour intensity depending on the culture conditions (Figure 1). The roots grown in the NSB bioreactor were the lightest, while those grown in the PlantForm TIS bioreactor were dark green.

**Figure 2:**
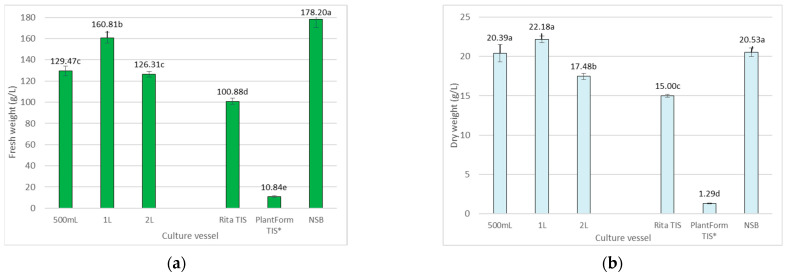
The fresh weight (FW) (**a**) and dry weight (DW) (**b**) (in g per liter) of *R. cartahmoides* transformed roots cultured in liquid WPM medium after 35 days. * The cultures were grown for 21 days. The results are expressed as means ± standard error (SE). There is no difference (*p* < 0.05) among the means marked with the same letter.

**Figure 3:**
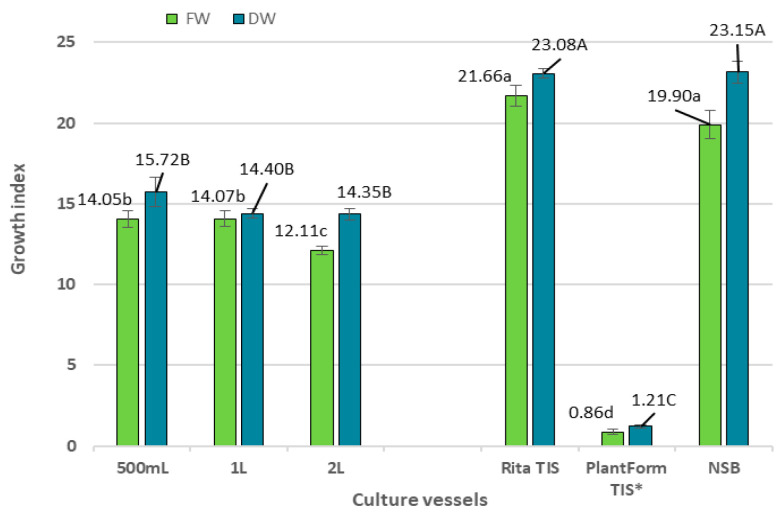
The growth index (Gi) for FW and DW of *R. cartahmoides* transformed roots cultured in liquid WPM medium after 35 days. * The cultures were grown for 21 days. The results are expressed as means ± standard error (SE). Means marked with the same uppercase or lowercase letter are not significantly different (*p* < 0.05).

In the present study, significant differences in biomass accumulation, i.e., fresh (FW) and dry weight (DW), were observed after five weeks, depending on the culture system (Figure 1 and Figure 2). The NSB bioreactor provided the optimal condition for the growth of *R. carthamoides* transformed roots. In this case, after one growth cycle, 178.2 g FW and 20.53 g DW were obtained (Figure 2); this represented approximately a 20-fold increase in the starting fresh weight and 23 times the initial inoculum dry weight (Figure 3). A similar growth index (Gi) was noted for roots cultured in the Rita^®^ TIS bioreactor; however, in this case, the roots demonstrated 1.5 times lower biomasses (100.88 g/L FW and 15 g/L DW) compared to the NSB bioreactor (Figure 2 and Figure 3).

The least favourable growth conditions for *R. carthamoides* transformed root growth were observed in the PlantForm TIS bioreactor: only 10.84 g/L FW and 1.29 g/L DW were achieved. It is worth emphasizing that the roots darkened and died after three weeks of growth in in vitro cultures. The PlantForm bioreactor was also found to be unsuitable for cultivating transformed roots of *Agastache rugosa* [20] or *Salvia austriaca* [21]. For example, *S. austriaca* transformed roots achieved more than three times lower DW than the cultures kept in 300 mL shaken Erlenmeyer flasks and mist-tricking bioreactor. Such low root biomass weight could be caused by shear forces, which cause mechanical damage to the fragile roots and inhibit their growth [21].

Transformed root cultures grown in bioreactors can lower cultivation costs by providing optimal conditions for high biomass yields and specialized metabolite production; the process can also be automated. Studies have evaluated the potential of numerous bioreactor types for root cultivation, e.g., bubble column, stirred tank, air-lift, and mist bioreactors or bioreactors with temporary immersion systems [7,22]. Among these, temporary immersion systems (TIS) are widely used in plant biotechnology. Although Rita^®^ bioreactors were designed for plant micropropagation, they can also be successfully used for root cultivation [19,20,23,24,25,26], as confirmed by the present study.

In TIS bioreactors, the plant materials are periodically flooded with a medium, with the plant tissue being immersed in a liquid medium, dried, and exposed to a gaseous environment in alternating cycles. In this case, it is important to determine the optimal length of immersion to ensure optimal humidity conditions and nutrient access. The immersion period usually lasts a few minutes, while exposure to gases can be up to several hours. TIS bioreactors provide ventilation and thus prevent ethylene and carbon dioxide accumulation in the culture vessel [22]. In the TIS system, the frequency and duration of immersion have a significant influence on the increase in biomass and the concentration of the specialized metabolites [27]. Longer intervals of immersion may positively influence cell growth, which takes up more oxygen when not frequently immersed; it may also allow more efficient respiration and greater production of ATP used for growth. On the other hand, longer intervals may cause water stress, enhancing the biosynthesis of specialized metabolites [28].

The most favourable conditions for *R. carthamoides* transformed root growth were noted for the NSB bioreactor (Figure 1, Figure 2 and Figure 3), when the growth medium was dispersed into tiny droplets sprayed onto the plant materials. This cultivation system improves the availability of medium ingredients and oxygen transfer and reduces shear stress. In the present study, *R. carthamoides* transformed roots cultured in the NSB bioreactor occupied the entire space within the vessel and demonstrated numerous lateral branches and root lengths reaching up to 10 cm (Figure 1). Several studies have also found this type of bioreactor to be suitable for cultivating transformed roots from various other plant species, including *Agastache rugosa* [20], *Leonurus sibiricus* [29], and *Panax quinquefolium* [30].

The growth of *R. carthamoides* transformed roots in shaken Erlenmeyer flasks was also satisfactory compared to the NSB bioreactor (Figure 2). The DW ranged from 17.48 g/L to 22.18 g/L, reaching the highest value in 1 L flasks. In this case, an approximately 15-fold increase in biomass over the initial inoculum was observed. Scaling up from 1 L to 2 L lowered the biomass of *R. cartahmoides* transformed roots (Figure 2). Similarly, AtAP1 transgenic roots of *Leonurus sibircus* demonstrated less growth in 3 L and 5 L flasks than in the 1 L shaken flask [29]. On the other hand, no differences in biomass production were observed for the transformed roots of *Astragalus membranaceus* [31] and *Cichorium intybus* var Orchies [32]. In contrast, Khalit et al. [33] demonstrated that the growth of *Withania somnifera* and *Nicotiana tabacum* transformed roots increased with the volume of the flask and the nutrient medium, which could be related to the increased absorption of oxygen: an important factor during root growth.

Several other parameters, such as growth-medium volume and shaking speed, influence the growth of roots in flasks. For example, *R. carthamoides* transformed roots demonstrated two-fold higher DW in 50 mL of medium (29 g/L) [13], compared to 80 mL (12 g/L) in 300 mL shaken Erlenmeyer flasks [12]. Jeong et al. [34] proposed that reducing the volume of the liquid medium in the flask can increase the gas exchange. Moreover, the air-to-liquid volume ratio in flask cultures can affect gas–liquid and liquid–solid mass transfer. Root growth may also be influenced by agitation speed, which may cause hydrodynamic stress. As Bernard et al. [32] demonstrated, *Cichorium intybus* var Orchies transformed roots achieved higher fresh weight when grown in 100 mL of culture medium at a speed of 90 rpm compared to those cultured in 400 mL of medium at 80 rpm. However, no change in root growth was noted for roots cultured at an increased agitation speed in 200 mL. Similarly, Thanonkeo et al. [35] noted that *Pueraria candollei* var. *mirifica* transformed roots achieved similar biomasses when cultured at different agitation speeds (90–130 rpm). Although the speed did not affect root biomass, a lower shaking speed was more beneficial for daidzein production.

The main difference between shaken flask and bioreactor culture is that in the former, the roots are immersed in the medium throughout the entire growth period. In contrast, in bioreactors, the medium is supplied in appropriate cycles, which affects inter alia the delivery of nutrients and oxygen to the roots. More efficient oxygen transport contributes to more efficient gas exchange, reduced oxygen limitations, and thus a lower occurrence of physiological disorders [22]. The solubility of oxygen decreases with increasing temperature and solute concentration. The complex structure of the roots may prevent the transport of undissolved gases, which generates almost anaerobic conditions [36].

### 2.2. Production of Caffeoylquinic Acid Derivatives and Flavonoids

**Figure 4:**
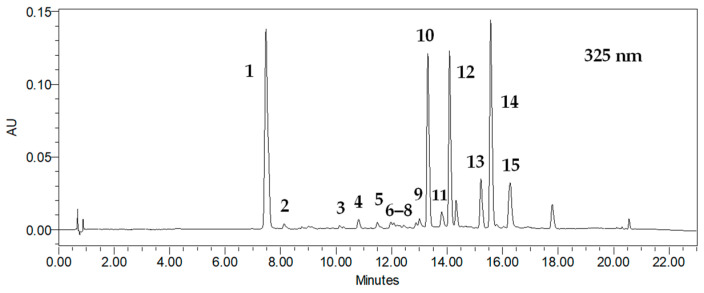
The HPLC-UV chromatogram of 80% (*v*/*v*) aqueous methanol extracts of *R. carthamoides* transformed roots cultured in liquid WPM medium for 35 days in the NSB bioreactor recorded at 325 nm. The identified peaks are as follows: (**1**) 5-*O*-caffeoylquinic acid (5-CQA, chlorogenic acid, CHA); (**2**) 4-*O*-caffeoylquinic acid (4-CQA); (**3**) 1,3-*O*-dicaffeoylquinic acid (1,3-diCQA); (**4**) quercetagetin hexoside; (**5**) quercetin hexoside 1; (**6**) quercetin hexoside 2; (**7**) luteolin hexoside; (**8**) patuletin hexoside; (**9**) 3,4-*O*-dicaffeoylquinic acid (3,4-diCQA); (**10**) 3,5-*O-*dicaffeoylquinic acid (3,5-diCQA); (**11**) 1,5-*O*-dicaffeoylquinic acid (1,5-diCQA); (**12**) 4,5-*O-*dicaffeoylquinic acid (4,5-diCQA); (**13**) 1,4,5-*O*-tricaffeoylquinic acid (1,4,5-triCQA); (**14**) tricaffeoylquinic acid 1 (tri-CQA 1); (**15**) tricaffeoylquinic acid 2 (tri-CQA 2).

HPLC-PDA analysis confirmed that *R. carthamoides* transformed roots accumulated eight CQAs, representing mono-CQA, di-CQA, and tri-CQA derivatives. This finding is consistent with our previous studies [12,13]. In addition, two tentatively identified tri-CQA derivatives and five flavonoid monoglycosides were revealed (Figure 4).

The specialized metabolites were identified by UPLC-PDA-ESI-MS^3^ assay (Table S1), as detailed by Skała et al. [12].

**Figure 5:**
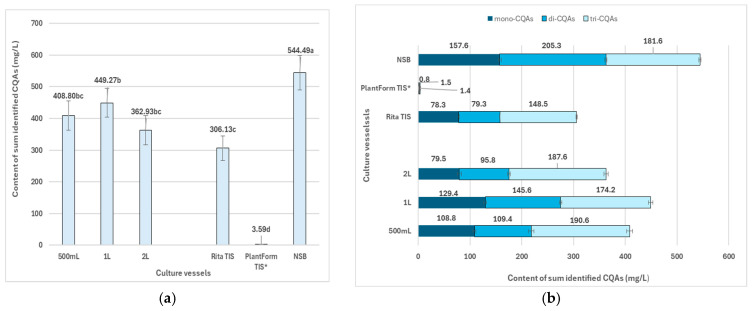
Productivity of total identified CQA derivatives (mg/L medium) (**a**) in *R. cartahmoides* transformed roots cultured in liquid WPM medium after 35 days. (**b**) The proportion of the content of CQA derivatives (mg/L medium). * The cultures were grown for 21 days. The results are expressed as means ± standard error (SE). Means marked with the same letter are not significantly different (*p* < 0.05).

A crucial consideration when obtaining material from in vitro plant cultures is the economic efficiency of the culture; therefore, to select the optimum conditions for effective specialized metabolite production, our analysis included both increases in the dry weight per liter of medium and compound concentration. After a 35-day growth period, the yield of total CQAs from the transformed roots of *R. carthamoides* varied between 3.6 mg/L and 544.5 mg/L, depending on the culture vessel used, with the highest level occurring in roots cultured in the NSB bioreactor (Figure 5a). The transformed roots in the NSB bioreactor also exhibited the highest biomass production, reaching 20.53 g/L DW (Figure 2b). In spray bioreactors, the medium is supplied to the culture in mist form, which prevents the shear forces that can damage the roots and significantly facilitates oxygen access to the cells due to the higher solubility of oxygen in air compared to liquid medium [37].

*R. carthamoides* transformed roots grown in shaken Erlenmeyer flasks of 1 L and 0.5 L volumes also demonstrated high capacities for CQA production, yielding 449.3 mg/L and 408.8 mg/L, respectively. This was followed by a 2 L flask (362.9 mg/L), Rita^®^ TIS (306.1 mg/L), and finally PlantForm TIS (3.6 mg/L) (Figure 5a). The results indicate that cultivation in TIS bioreactors inhibited the accumulation of all specialized metabolites identified in *R. carthamoides* transformed roots (Figure 5).

Specifically, the PlantForm TIS bioreactor was not beneficial for cultivating *R. carthamoides* transformed roots and producing specialized metabolites, as these roots only biosynthesized 3.6 mg/L of CQAs (Figure 5a). Similarly, in a previous study, *Agastache rugosa* transformed roots growing in the PlantForm TIS bioreactor also exhibited the lowest concentration of specialized metabolites, slowest growth, and lowest biomass increments; they also demonstrated a poorer phytochemical profile compared to those grown in the nutrient sprinkle bioreactor [20]. This may have been caused by inappropriate culture conditions. Two key factors affecting secondary metabolite concentration and biomass increase in the TIS system are believed to be the frequency and duration of immersion. Insufficient intervals between immersions may lead to oxygen deficiency and low respiration levels (inhibiting oxygen absorption), resulting in reduced ATP levels and slower culture growth [28]. Conversely, too frequent immersion may generate excessive shear forces, causing mechanical damage to the roots. Manuhara et al. [28] noted that longer immersion intervals increased saponin concentration in *Talinum paniculatum* adventitious roots. Similarly, *Gynura procumbens* adventitious roots [24] and shoots [38] exhibited higher levels of flavonoids when subjected to extended duration immersions. As Manuhara et al. [28] suggested, it is likely that there was an optimal concentration of oxygen and nutrients in those conditions. Another possibility is that stress associated with water deficiency may increase the concentration of specialized metabolites. Further research is required to optimize parameters such as immersion time and immersion frequency or inoculum size for the growth of *R. carthamoides* transformed roots and the production of specialized metabolites.

Although no qualitative differences were noted between *R. carthamoides* transformed roots cultured in various culture systems, they did display variation in the proportions of CQAs (Figure 5b). Roots grown in shaken Erlenmeyer flasks, regardless of flask size, and in the Rita^®^ TIS bioreactor accumulated the largest amounts of tri-CQAs, which made up 39–52% of the total CQA content. In contrast, roots cultured in the NSB bioreactor predominantly accumulated di-CQAs (Figure 5b), while the roots of three-year-old soil-grown plants were primarily composed of mono-CQAs, with chlorogenic acid being the most prominent component [12].

**Figure 6:**
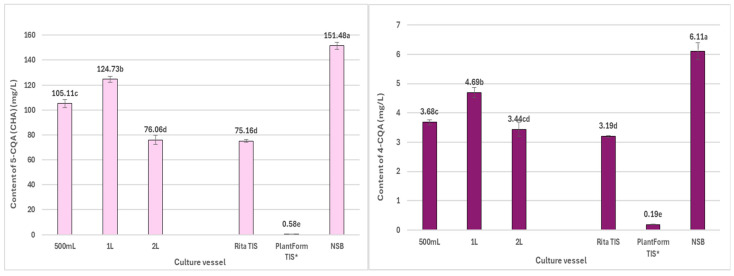
Productivity of individual mono-CQAs (mg/L medium) in *R. cartahmoides* transformed roots cultured in liquid WPM medium after 35 days. * The cultures were grown for 21 days. The compound codes refer to those applied below Figure 4. The results are expressed as means ± standard error (SE). Means marked with the same letter are not significantly different (*p* < 0.05).

**Figure 7:**
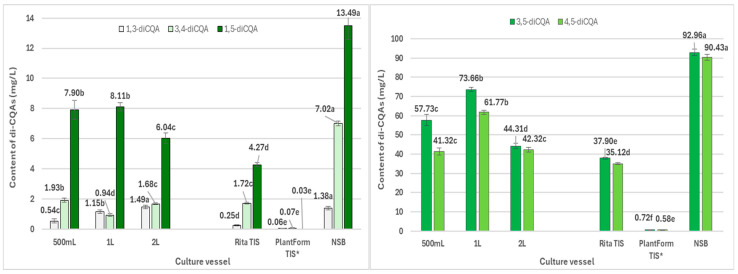
Productivity of individual di-CQAs (mg/L medium) in *R. cartahmoides* transformed roots cultured in liquid WPM medium after 35 days. * The cultures were grown for 21 days. The compound codes refer to those applied below Figure 4. The results are expressed as means ± standard error (SE). Means marked with the same letter are not significantly different (*p* < 0.05).

**Figure 8:**
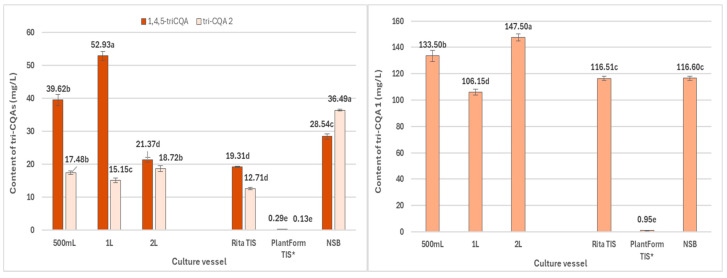
Productivity of individual tri-CQAs (mg/L medium) in *R. cartahmoides* transformed roots cultured in liquid WPM medium after 35 days. * The cultures were grown for 21 days. The compound codes refer to those applied below Figure 4. The results are expressed as means ± standard error (SE). Means marked with the same letter are not significantly different (*p* < 0.05).

In the present study, the quantitatively dominant CQAs in *R. carthamoides* transformed roots were 5-*O*-caffeoylquinic acid (5-CQA, chlorogenic acid, compound **1**, t_R_ = 7.2 min) and the tentatively identified tri-CQA derivative 1 (t_R_ = 15.6 min) (**14**) (Figure 6, Figure 7 and Figure 8). The highest level of 5-CQA was noted in the roots cultivated in the NSB bioreactor (7.38 mg/g DW; 151.5 mg/L) (Table S2 and Figure 6), while tri-CQA derivative 1 predominated in the roots from the 2 L shaken Erlenmeyer flask (8.44 mg/g DW; 147.5 mg/L) (Table S2 and Figure 8).

The roots grown in the NSB bioreactor also yielded the highest amounts of 3,5-*O*-dicaffeoylquinic acid (3,5-diCQA) (**10**) (4.53 mg/g DW; 92.96 mg/L), 4,5-*O*-dicaffeoylquinic acid (4,5-diCQA) (**12**) (4.41 mg/g DW; 90.43 mg/L), 1,5-*O*-dicaffeoylquinic acid (1,5-diCQA) (**11**) (0.66 mg/g DW; 13.49 mg/L), 3,4-*O*-dicaffeoylquinic acid (3,4-diCQA) (**9**) (0.34 mg/g DW; 7.02 mg/L) (Table S2; Figure 7), and 4-*O*-caffeoylquinic acid (4-CQA) (**2**) (0.30 mg/g DW; 6.11 mg/L) (Table S2 and Figure 6). Among the tri-CQAs, the highest level of 1,4,5-triCQA (**13**) was detected in roots from 1 L Erlenmeyer flasks (2.39 mg/g DW; 52.9 mg/L) (Table S2 and Figure 8). Previous studies have noted that CQAs may be successfully accumulated in transformed roots of different plant species, such as *Cichorium intybus*, *Lactuca indica*, *L. virosa*, or *Leonurus sibiricus* [14]. The best source of these phenolic acids is believed to be *C. intybus* root [39].

The present research demonstrates that a single culture cycle in a bioreactor can yield about 15 times higher levels of CQAs (544.5 mg) from *R. carthamoides* transformed roots in just 35 days, compared to 35 mg/g DW obtained from three-year-old soil-grown plants [12].

In conclusion, *R. carthamoides* transformed roots provide a sustainable source of CQAs without harming natural plant populations or crops. They are genetically homogeneous, free from environmental contamination, and can be cultivated regardless of the season or climate zone. *R. carthamoides* is endemic to the Altai and Sayan Mountains of Siberia, Mongolia, Kazakhstan, and China, where it is typically found in alpine and subalpine meadows and tundra at elevations of 1850–2000 m above sea level [9,10,11,40]. Unfortunately, *R. carthamoides* is an endangered species due to overgrazing by livestock and extensive harvesting for medicinal purposes [18,41].

**Figure 9:**
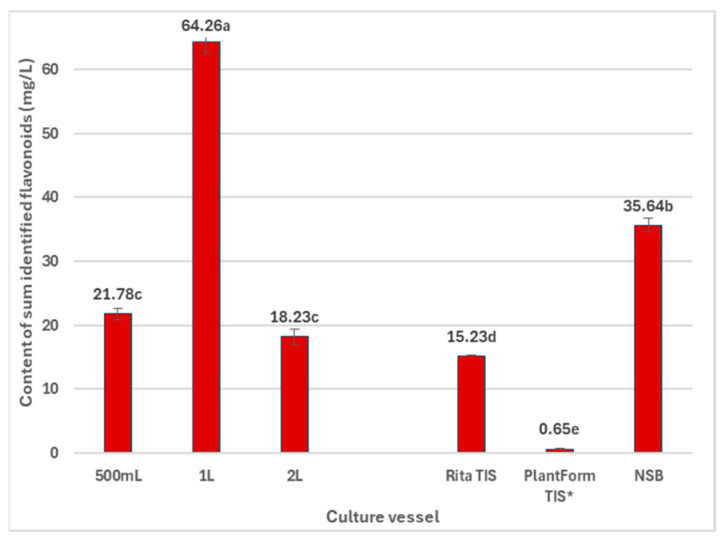
Productivity of total identified flavonoid monoglycosides (mg/L medium) in *R. cartahmoides* transformed roots cultured in liquid WPM medium after 35 days. * The cultures were grown for 21 days. The results are expressed as means ± standard error (SE). Means marked with the same letter are not significantly different (*p* < 0.05).

**Figure 10:**
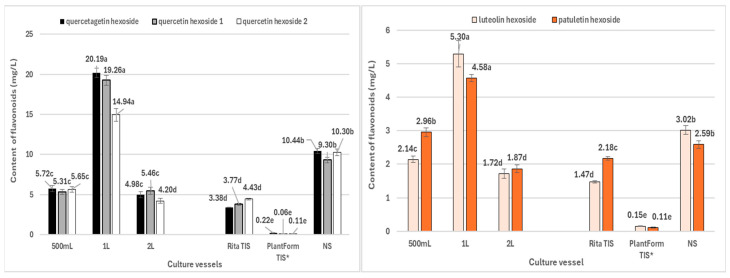
Flavonoid monoglycoside productivity (mg/L medium) in *R. cartahmoides* transformed roots cultured in liquid WPM medium after 35 days. * The cultures were grown for 21 days. The compound codes refer to those applied below Figure 4. The results are expressed as means ± standard error (SE). Means marked with the same letter are not significantly different (*p* < 0.05).

The most efficient system for flavonoid production in *R. carthamoides* transformed roots was cultivation in 1 L Erlenmeyer flasks, yielding 64.3 mg/L (2.90 mg/g DW) (Figure 9 and Figure 10, Table S2). The primary compounds were quercetagetin hexoside (**4**) and quercetin hexoside 1 (**5**) (Figure 10). Notably, the roots from soil-grown *R. carthamoides* do not biosynthesize flavonoids [12].

**Figure 11:**
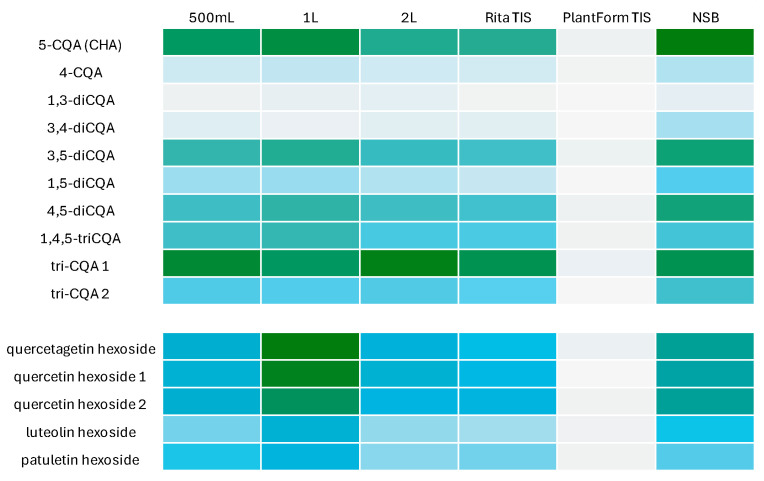
Heat map visualization of CQA derivative and flavonoid productivity (mg/L). The colour intensity of boxes indicates the level of specialized metabolites; darker green indicates high content while gray indicates low content. The compound codes refer to those applied below Figure 4.

The trends in CQAs and flavonoid content in *R. carthamoides* transformed roots cultured in various culture systems, including shaken Erlenmeyer flasks, temporary immersion systems, and nutrient sprinkle bioreactors, are also illustrated in a heat map (Figure 11).

The observed differences in the content of specialized metabolites in *R. carthamoides* transformed roots in different culture systems can be attributed to several factors. The primary distinction between flask and bioreactor culture lies in the continuous shaking of the roots in flasks, which may induce hydromechanical stress. Moreover, in flasks, the roots remain submerged in the medium throughout the growth period, whereas in bioreactors, the nutrient medium is delivered in cycles; the latter allows improved nutrient and oxygen transport to the roots. Enhanced oxygen supply facilitates better gas exchange, reduces oxygen limitations, and ultimately minimizes physiological disorders [22].

### 2.3. Antioxidant Properties of R. carthamoides Transformed Roots

**Figure 12:**
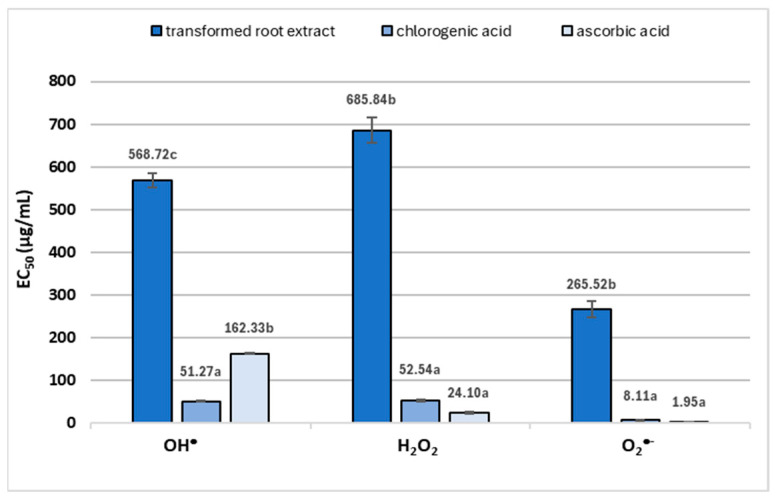
Antioxidant activity of *R. carthamoides* transformed root. Radical-scavenging efficiency is expressed as EC_50_, which represents the effective concentration of the plant material or reference standard (µg/mL) required to reduce the initial concentration of OH^•^, H_2_O_2_, or O_2_^•−^ by 50%. The results are expressed as means ± standard error (SE). Means marked with the same letter are not significantly different.

The phytochemical composition of *R. carthamoides* transformed roots cultured in the NSB bioreactor was compared with the results of three in vitro antioxidant activity assays testing the ability to scavenge hydroxyl radicals (OH^•^), reduce hydrogen peroxide (H_2_O_2_), or inactivate superoxide radicals (O_2_^•−^). The extract from *R. carthamoides* transformed roots displayed a concentration-dependent capacity to scavenge all target radicals, with total activity scores varying widely: EC_50_ = 265.5–685.8 µg/mL (Figure 12). These findings are consistent with those of previous studies [42,43] indicating that *R. carthamoides* root extracts possess antioxidant properties in basic chemical tests in non-biological environments (DPPH, ABTS, and FRAP) and in models using human blood plasma.

The antioxidant activity of the transformed roots examined in the present study can primarily be attributed to chlorogenic acid (CHA) (EC_50_ = 8.1–52.5 µg/mL), one of the dominant polyphenols in the extract (Figure 6), which is used as a marker for the plant material activity. According to the literature [44], CHA and its isomers are potent antioxidants; this is due to their structure, which includes one to two aromatic rings linked with hydroxyl groups. The one-electron oxidation products of these acids, formed during reactions with free radicals, decompose rapidly into non-radical products. Numerous in vitro tests have shown that CHA isomers can scavenge various radicals, including DPPH, ABTS, superoxide anion (O_2_^•−^), and hydroxyl radical (OH^•^), and inhibit both lipid oxidation and peroxynitrite (ONOO^−^) [44]. Therefore, it can be hypothesized that the scavenging capacity of *R. carthamoides* root extracts for in vivo-relevant radicals is dependent on polyphenols, particularly the dominant isomers and derivatives of CHA.

## 3. Materials and Methods

### 3.1. Plant Material

The transformed roots of *R. carthamoides* were obtained by *Rhizobium rhizogenes* transformation of the leaves of four-week-old shoots obtained from aseptic seedlings, as detailed previously [12]. The voucher specimen of the plant (S_RC_014) was deposited at the Department of Biology and Pharmaceutical Botany, Medical University of Lodz (Lodz, Poland). *R. carthamoides* transformed roots were cultured in hormone-free liquid full-strength Woody Plant medium (WPM) [45] (Duchefa Biochemie B.V., Haarlem, The Netherlands) supplemented with 3% sucrose on a rotary shaker at 80 rpm, in light conditions (16/8 h light/dark photoperiod; PPFD of 40 µmol m^−2^ s^−1^ under a cool-white fluorescent lamp). These culture conditions were selected as optimal for root growth based on previous experiments [12,13]. *R. carthamoides* transformed roots were subcultured every five weeks.

### 3.2. R. carthamoides Transformed Roots Cultivation

The roots were grown in Erlenmeyer flasks of different volumes (500 mL, 1 L, and 2 L) and two types of bioreactor systems: (a) a commercially available temporary immersion system (TIS) Rita^®^ (Récipient à Immersion Temporiaire Automatiqe, Vitropic, St-Mathieu de Tréviers, France) and PlantForm (PlantForm AB, Hjärup, Sweden) and (b) a nutrient sprinkle 5 L bioreactor (NSB). The Erlenmeyer flasks were supplemented with 150 mL, 300 mL, and 600 mL of medium (500 mL, 1 L, and 2 L flasks, respectively) and were cultured on a rotary shaker at 80 rpm. A 1 L Rita^®^ bioreactor contained 350 mL of a medium delivered by a DT4.4 pressure pump (Gebr. Becker GmbH, Wuppertal, Germany) with a capacity of 4.2 m^3^/h and pressure of 1000 mbar. The PlantForm bioreactor, 180 mm (length) × 150 mm (width) × 150 mm (height), contained 500 mL of medium supplied by air via Hailea pumps (Guangdong Hailea Group Co., Ltd., Chaozhou, China). The root cultivation in both TIS bioreactors was performed with an immersion frequency of 10 min of flooding for every 70 min of standby period. This operation was controlled by a temporary electric relay.

A 5L NSB bioreactor consists of two parts: a main container, in the upper part, where root growth occurs, and the bottom part that serves as a reservoir for 1 L of medium, as previously described by Piątczak et al. [46]. The medium was provided to the spray nozzle by a peristaltic pump every 1.5 min with a sprinkling time of 40 s.

Subcultures were carried out every five weeks by transferring 1.290 ± 0.006 g/flask FW to a 0.5 L flask, 3.202 ± 0.224 g/flask to a 1 L flask, 5.780 ± 0.093 g/flask to a 2 L flask, 1.558 ± 0.025 g/bioreactor to Rita^®^, 2.907 ± 0.129 g/bioreactor to PlantForm, and 8.527 ± 0.237 g/bioreactor to NSB bioreactor. All cultures were maintained in light conditions (16 h/8 h light/dark photoperiod; PPFD of 40 µmol m^−2^ s^−1^ under cool-white fluorescent lamps).

The fresh weight (FW) and the dry weight (DW) (after lyophilization using freeze dryer Alpha 1-2 LDPlus; M. Christ Gefriertrocknungsanlagen, Osterode am Harz, Germany) were calculated and expressed in g/L. In addition, the growth index (Gi) was also determined by the following formula: final FW (or DW) − initial FW (or DW)/initial FW (or DW). The experiment was repeated three times. In the case of root cultivation in TIS bioreactors, the experiments were performed six times.

### 3.3. Production of Caffeoylquinic Acid Derivatives and Flavonoids

#### Extract Preparation and Determination of Secondary Metabolites by UHPL-PAD Analysis

The CQAs and flavonoid glycosides were identified by UPLC-PDA-ESI-MS^3^ and by comparison of their UV-Vis spectra and MS data (Supplementary Materials), as described previously [12]. The crude hydromethanolic extracts (80% *v*/*v*) from the lyophilized transformed roots (500 mg) were obtained through three extractions of plant materials; once with 25 mL and then twice with 10 mL of 80% (*v*/*v*) aqueous methanol, at 35 °C for 15 min, using an ultrasonic bath (InterSonic IS-20, Olsztyn, Poland). The quantitative analysis was conducted using HPLC-PDA (Supplementary Materials), as described previously [12]. The chlorogenic acid isomers, dicaffeoylquinic acid isomers and tricaffeoylquinic acid and its derivative, and flavonoid monoglycosides were quantified as equivalents of chlorogenic acid, cynarin, and isoquercitin, respectively. The level of the specialized metabolites was expressed in mg/g DW (Table S2) and as yield in mg per liter of WPM medium (mg/L) (Figure 5, Figure 6, Figure 7, Figure 8, Figure 9, Figure 10 and Figure 11).

### 3.4. Antioxidant Activity

#### 3.4.1. Reagents

High-purity reagents for spectrophotometric analyses, including salicylic acid, iron (II) sulphate heptahydrate, hydrogen peroxide, horseradish peroxidase, 4-aminoantipyrine (4-AAP), phenol, xanthine, xanthine oxidase from bovine milk, nitrotetrazolium blue chloride (NBT), and ascorbic acid (AA), were purchased from SigmaAldrich (Seelze, Germany/St. Louis, MO, USA); chlorogenic acid (CHA) was obtained from Phytolab (Vestenbergsgreuth, Germany); phosphate-buffered saline (PBS) was purchased from Biomed (Lublin, Poland).

#### 3.4.2. Extract Preparation

The transformed roots cultured in the NSB bioreactor (3.5 g) were lyophilized, powdered, and extracted with 80% (*v*/*v*) aqueous methanol according to the procedure described above.

#### 3.4.3. Antioxidant Analysis

The antioxidant activity of the transformed roots of *R. carthamoides* and chlorogenic acid (CHA) towards common in vivo-relevant oxidants was evaluated in vitro using three cell-free assays: the OH^•^ radical reduction test, where the radical was released by the Fenton reaction and its concentration was monitored by reaction with salicylic acid [47]; the H_2_O_2_ reduction test, involving reaction with 4-AAP and phenol, catalyzed in the presence of horseradish peroxidase [47]; and the O_2_^•−^ reduction test, where the radical, released in the xanthine oxidation, was monitored through the NBT reduction reaction [47]. The results were presented as EC_50_ values, defined as the concentration of the analyte that reduces the initial amount of the oxidant by 50%. These values were derived from concentration-scavenging curves. Ascorbic acid (AA) standard was used as a positive control.

### 3.5. Statistical Analysis

All results were calculated as means ± SE. The Shapiro–Wilk test was used to evaluate the normality of the data. Statistical comparisons were carried out using one-way analysis of variance (ANOVA) with Tukey’s post hoc test (*p* < 0.05) (Statistica 13.1 software, StatSoft, Krakow, Poland). A heat map visualization was created using Microsoft Office Excel.

## 4. Conclusions

Cultivating *R. carthamoides* transformed roots in a nutrient sprinkle bioreactor is an effective system for intensive biomass and CQA derivative production. In a single-cycle system, after 35 days, it is possible to obtain approximately 15-fold greater amounts of CQAs from a single bioreactor than from three-year-old soil-grown plants. This system is highly beneficial for obtaining valuable metabolites, offering an alternative source for these compounds. This biotechnological approach can help reduce the overexploitation of *R. carthamoides* in its natural habitat, where it is currently threatened.

## Data Availability

Data are contained within the article.

## Supplementary Materials

The following supporting information can be downloaded at: https://www.mdpi.com/article/10.3390/ijms26041422/s1.

## Abbreviations

The following abbreviations are used in this manuscript:

CHA	Chlorogenic acid; 5-*O*-caffeoylquinic acid
CQA	Caffeoylquinic acid derivative
NSB	Nutrient sprinkle bioreactor
TIS	Temporary immersion system
WPM	Woody Plant medium
