# Supplementary material for: The Enhancement of Biomass Accumulation, Caffeoylquinic Acid Derivative Production, and Antioxidant Activity of Rhaponticum carthamoides Transformed Roots Cultured in a Nutrient Sprinkle Bioreactor
Source: Int J Mol Sci. 2025 Feb 8;26(4):1422. doi: 10.3390/ijms26041422 (PMC11855349; doi:10.3390/ijms26041422)
Supplement: Supplementary file 1 [file ijms-26-01422-s001.zip › ijms-3437585-supplementary.pdf]

Article

The Enhancement of Biomass Accumulation, Caffeoylquinic Acid Derivative Production, and Antioxidant Activity of *Rhaponticum carthamoides* Transformed Roots Cultured in a Nutrient Sprinkle Bioreactor

Ewa Skała, Monika A. Olszewska, and Agnieszka Kicel

Materials and Methods

Qualitative UHPLC-PDA-ESI-MS^3 profiling of polyphenols in the *R. carthamoides* transformed roots extract

The UPLC-PDA-ESI-MS^3 analysis was conducted on a UPLC-3000 RS system (Dionex, Germany), equipped with a dual low-pressure gradient pump, an autosampler, a column compartment, a diode array detector, and an AmaZon SL ion trap mass spectrometer interfaced with an electrospray ionization (ESI) source (Bruker Daltonik, Germany). Chromatographic separation was achieved using a Kinetex XB-C18 column (1.7 µm, 150 × 2.1 mm i.d., Phenomenex, USA). The mobile phase consisted of solvent A (0.1% formic acid in aqueous solution, v/v) and solvent B (acetonitrile containing 0.1% formic acid, v/v). The gradient elution profile was as follows: 0-45 minutes, 6-26% B (v/v); 45-55 minutes, 26-95% B; 55-63 minutes, 95% B; and 63-70 minutes, 95-6% B. The flow rate was maintained at 0.3 mL/min, and the column temperature was kept constant at 25°C. UV-Vis spectra were recorded over 200-600 nm, and chromatograms were acquired at wavelengths of 245, 325, and 350 nm. The LC eluate was directly introduced into the ESI interface without splitting. The mass spectrometric conditions were as follows: nebulizer pressure of 40 psi, dry gas flow rate of 9 L/min, dry temperature of 300°C, and capillary voltage of 4.5 kV. The analysis was performed in negative ion mode with a scan range from m/z 200 to 2200.

Quantitative HPLC-PDA analysis of polyphenols in the *R. carthamoides* transformed roots extract

The HPLC-PDA analysis was carried out on a Waters 600E Multisolvent Delivery System (Waters, USA), equipped with a PDA detector (Waters 2998) operating within the wavelength range of 220–450 nm. A model 7725 sample injection valve (Rheodyne, CA, USA) with a 5 µL injection loop was used, and data collection was performed using a LC workstation with Waters Empower 2 software. The chromatographic separation was achieved on a C18 Ascentis Express analytical column (2.7 µm, 75 mm × 4.6 mm i.d.; Supelco, PA, USA), protected by a C18 Ascentis C18 Supelguard column (3 µm, 20 mm × 4 mm i.d.; Supelco). The mobile phase consisted of solvent A (0.5% aqueous orthophosphoric acid, v/v) and solvent B (acetonitrile), with the following elution gradient: 0-1 min, 5% B (v/v); 1–16 min, 5–30% B; 16–17 min, 30–50% B; 17–19 min, 50% B; 19–20 min, 50–5% B; and 20–25 min, 5% B (for equilibration). The flow rate was set at 1.4 mL/min, and the column temperature was maintained at 30°C.

Results

Table S1. The UV-Vis and UHPLC-PDA-ESI-MS^3 data of identified polyphenols in the *R. carthamoides* transformed roots extract

No.[a]	Compound	UV (nm)	$[M-H]^-$ *m/z*	MS^2 (% relative abundance)	MS^3 (% relative abundance)
1	5-*O*-caffeoylquinic acid (5-CQA, CHA)	325	353	191(100); 179(3.5);	171(26); 127(16); 111(23); 85(100)
2	4-*O*-caffeoylquinic acid (4-CQA)	325	353	191(55); 179(44); 173(100)	
3	1,3-*O*-dicaffeoylquinic acid (1,3-diCQA)	328	515	353(100); 335(25); 191(10); 179(20);	191(100); 179(35); 135(5)
4	Quercetagetin hexoside	356	479	385(8),317(100)	
5	Quercetin hexoside 1	350	463	301(100); 179(5)	273(23); 257(17); 179(100); 151(83)
6	Quercetin hexoside 2	350	463	301(100)	
7	Luteolin hexoside	349	447	285(100)	241(97); 223(58); 213(36); 175(100); 151(66)
8	Patuletin hexoside	365	493	331(100); 316(4.5)	316(68); 287(4)
9	3,4-*O*-dicaffeoylquinic acid (3,4-diCQA)	325	515	353(100); 335(17); 317(19); 299(32); 255(14); 203(40)	191(55); 179(62); 173(100)
10	3,5-*O*-dicaffeoylquinic acid (3,5-diCQA)	328	515	353(100); 191(16)	191(100); 179(1.1)
11	1,5-*O*-dicaffeoylquinic acid (1,5-diCQA)	328	515	353(100); 191(3)	191(100); 179(33)
12	4,5-*O*-dicaffeoylquinic acid (4,5-diCQA)	328	515	353(100); 335(2.7); 299(10); 255(6.5); 203(16); 173(7)	191(29); 179(66); 173(100)
13	1,4,5-*O*-tricaffeoylquinic acid (1,4,5-triCQA)	325	677	515(20); 497(100); 353(30); 335(28)	353(100); 335(11); 191(6); 179(8)
14	Tricaffeoylquinic acid 1 (tri-CQA 1)	328	793	631(100); 614(41); 515(27);498(16)	515(63); 469(100); 353(71)
15	Tricaffeoylquinic acid 2 (tri-CQA 2)	328	793	631(100); 469(6); 353(2);	469(100); 451(34); 353(59); 191(10)

[a] peak number refer to Figure 4.

Table S2. The content of caffeoylquinic acid derivatives and flavonoids (mg/g DW) in *R. carthamoides* transformed roots cultured in liquid WPM medium for 35 days.

Compound	Flask	Bioreactor				
	500 mL1	1L	2L	Rita TIS	PlantForm TIS*	NSB
Content of caffeoylquinic acid derivatives (mg/g DW)						
5-CQA	5.154±0.150	5.624±0.109	4.352±0.218	5.012±0.062	0.452±0.004	7.379±0.145
4-CQA	0.180±0.004	0.211±0.008	0.197±0.014	0.213±0.001	0.144±0.009	0.297±0.014
1,3-diCQA	0.026±0.006	0.052±0.005	0.085±0.006	0.017±0.002	0.044±0.005	0.067±0.006
3,4-diCQA	0.094±0.007	0.043±0.004	0.096±0.003	0.114±0.004	0.057±0.005	0.342±0.007
3,5-diCQA	2.831±0.140	3.322±0.051	2.535±0.085	2.528±0.024	0.556±0.010	4.529±0.080
1,5-diCQA	0.387±0.031	0.366±0.012	0.346±0.020	0.285±0.009	0.023±0.004	0.657±0.045
4,5-diCQA	2.026±0.089	2.785±0.052	2.421±0.072	2.342±0.039	0.449±0.010	4.405±0.075
1,4,5-triCQA	1.943±0.083	2.387±0.063	1.222±0.042	1.288±0.014	0.224±0.010	1.391±0.039
tri-CQA 1	6.546±0.214	4.786±0.105	8.439±0.147	7.769±0.094	0.736±0.018	5.680±0.073
tri-CQA 2	0.857±0.030	0.683±0.032	1.071±0.051	0.847±0.024	0.101±0.005	1.778±0.016
Content of flavonoids (mg/g DW)						
Quercetagetin hexoside	0.280±0.019	0.910±0.026	0.285±0.023	0.225±0.004	0.170±0.010	0.509±0.015
Quercetin hexoside 1	0.261±0.016	0.869±0.028	0.312±0.026	0.252±0.007	0.048±0.005	0.453±0.017
Quercetin hexoside 2	0.277±0.017	0.674±0.036	0.240±0.018	0.296±0.007	0.088±0.007	0.502±0.020
Luteolin hexoside	0.105±0.005	0.239±0.018	0.098±0.008	0.098±0.002	0.116±0.007	0.147±0.006
Patuletin hexoside	0.145±0.007	0.206±0.005	0.107±0.006	0.145±0.003	0.084±0.011	0.126±0.006
Sum of flavonoids	1.068	2.898	1.043	1.015	0.507	1.736

*the cultures were grown for 21 days
